# Psychophysical therapy and underlying neuroendocrine mechanisms for the rehabilitation of long COVID-19

**DOI:** 10.3389/fendo.2023.1120475

**Published:** 2023-09-29

**Authors:** Qing-Tai Meng, Wu-Qi Song, Leonid P. Churilov, Feng-Min Zhang, Yu-Feng Wang

**Affiliations:** ^1^ WU Lien-Teh Institute, Department of Microbiology, Harbin Medical University, Harbin, China; ^2^ Department of Experimental Tuberculosis, St. Petersburg State Research Institute of Phthisiopulmonology, Saint-Petersburg, Russia; ^3^ Department of Physiology, Harbin Medical University, Harbin, China; ^4^ International Translational Neuroscience Research Institute, Zhejiang Chinese Medical University, Hangzhou, China

**Keywords:** sequelae, psychophysical therapies, hormone, rehabilitation, long COVID-19

## Abstract

With the global epidemic and prevention of the COVID-19, long COVID-19 sequelae and its comprehensive prevention have attracted widespread attention. Long COVID-19 sequelae refer to that three months after acute COVID-19, the test of SARS-CoV-2 is negative, but some symptoms still exist, such as cough, prolonged dyspnea and fatigue, shortness of breath, palpitations and insomnia. Its pathological mechanism is related to direct viral damage, immunopathological response, endocrine and metabolism disorders. Although there are more effective methods for treating COVID-19, the treatment options available for patients with long COVID-19 remain quite limited. Psychophysical therapies, such as exercise, oxygen therapy, photobiomodulation, and meditation, have been attempted as treatment modalities for long COVID-19, which have the potential to promote recovery through immune regulation, antioxidant effects, and neuroendocrine regulation. Neuroendocrine regulation plays a significant role in repairing damage after viral infection, regulating immune homeostasis, and improving metabolic activity in patients with long COVID-19. This review uses oxytocin as an example to examine the neuroendocrine mechanisms involved in the psychophysical therapies of long COVID-19 syndrome and proposes a psychophysical strategy for the treatment of long COVID-19.

## 1 Introduction

The coronavirus disease 2019 (COVID-19) has not only a high morbidity and mortality, but also long-lasting sequelae that affect multiple systems, constitute intense obstacle for normal daily life and work, and impose huge burden to the healthcare system. Many cohort studies have shown that following the acute phase of COVID-19, 40–90% of patients have chronic symptoms that last more than three weeks after recovering from active infection although they exhibit negative test results for SARS-CoV-2 in conventional throat and nose swab COVID test (1–4). If these symptoms last beyond 12 weeks after acquiring the infection, with no alternative diagnosis, the patients are considered as having a long COVID-19 (5, 6). However, the drug therapies administrated in managing the symptoms and rehabilitation of the long COVID-19 sequelae commonly have limited therapeutic effectiveness (7). The non-drug therapies, mainly psychophysical therapies were recently employed for the rehabilitation of long COVID-19. Psychophysical therapy involves various mechanisms, including promotion of cell metabolism, modulation of immune cell activity, anti-oxidation, and neuroendocrine regulation (8, 9). Notably, COVID-19 has been shown to significantly impact neuroendocrine function, with chemosensory dysfunction being one of the most common symptoms observed (10, 11). Based on this fact, we propose that psychophysical therapy utilizing neuroendocrine mechanisms may offer an effective approach to treat long COVID-19.

This article aims to explore the pathological processes and clinical manifestations of long COVID-19, the role and mechanisms of psychophysical therapy, and the potential use of oxytocin as an example of how neuroendocrine hormones can aid in the rehabilitation of long COVID-19 through psychophysical therapy.

## 2 The symptoms and pathogenesis of long COVID-19 sequelae

**Figure 1:**
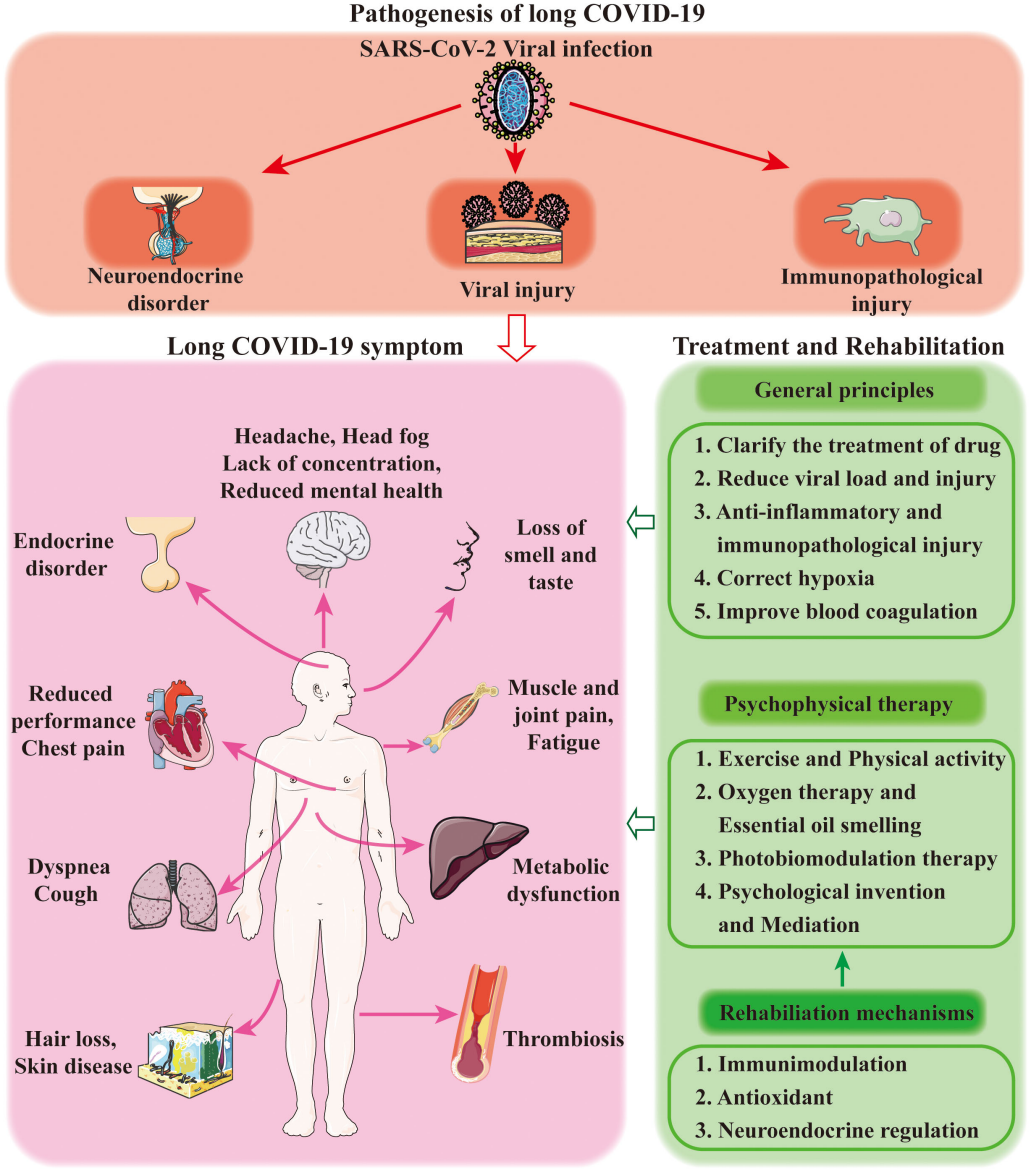
Diagrammatic summary of the common symptoms, pathogenesis and treatments of long COVID-19 sequelae.

People with long COVID-19 involve almost all the organ systems (12). The occurrence of long COVID-19 is closely associated with viral invasion and immunological injury, poor control of complications, cardiopulmonary and cerebral sequelae as well as social stress (13). The factors involving direct and indirect pathogenesis of SARS-CoV-2 and symptoms of long COVID-19 sequelae are summarized in **Figure 1**.

### 2.1 The viral infection and direct damage in long COVID sequelae

The SARS-CoV-2 infection causes prompt and direct damage to the tissues while SARS-CoV-2 RNA has been identified in fecal specimens of patients with COVID-19 during and beyond the acute phase (13). Virus-specific pathological variations specifically affect oxidative stress, immunological function, impaired diffusional O_2_ conductance and inflammation (14). Many symptoms of long COVID-19 occur or do not recover completely after the acute phase, including pulmonary fibrosis, endothelial damage, microvascular injury, brain fog, cardiac damage, muscle weakness, poor exercise tolerance and reduced sex steroid secretion and infertility (15). Moreover, different organ systems respond to SARS-CoV-2 infection differently. For example, in the cardiovascular system, alongside direct virus-induced injuries, inflammatory plaque instability and plaque rupture can contribute to myocardial infarction-induced long COVID-19 complications. Vascular leakage is related to SARS-CoV-2 induced clot formation in association with activation of Factor XIIa, complements and platelets, endothelial dysfunction, immune cell responses with cytokine-mediated action (16).Viruses and autoantibodies can damage the midbrain sympathetic center and change adrenergic and muscarinic neurotransmission, cause a hyper-adrenergic state and peripheral denervation, and result in blood pooling in the lower extremities and reflex tachycardia (17).In long COVID-19, the olfactory bulbs and taste buds are among the first organs infected by SARS-CoV-2, and thus anosmia and hypogeusia are the typical findings in the patients (18). Notably, anosmia and hypogeusia are also COVID-19-specific anxiety risk factors (19). In addition, long-intensive care syndrome and medical or clinical sequelae can contribute to long COVID-19 (20). Moreover, the reduced hypothalamic neural activity in long COVID-19 patients is likely because of olfactory bulb-mediated viral infection of the hypothalamus (21). It was identified that SARS-CoV-2 infection has significant longitudinal effects on greater changes in global brain structure, and a greater cognitive decline or the loss of sensory input due to anosmia in the patients (22).

### 2.2 The immuno-pathological injuries in long COVID sequelae.

Immune system dysregulation can manifest as hyperinflammation, cytokine storm, and immune-mediated multi-system damage. These effects can cause the aggregation of inflammatory cells and the release of pro-inflammatory and pro-fibrotic cytokines/factors (23). The inflammation can also cause secondary hemophagocytic lymphohistiocytosis, arthritis, skin psoriasis, systemic lupus erythematosus, Grave’s diseases, and immune thrombocytopenic purpura (24). Notably, there are at least 14 pentapeptides shared by the SARS-CoV-2 S-protein, thyroid, pituitary, adrenal cortex autoantigens and beta-cells of the islets of Langerhans. They all belong to the immunoreactive epitopes of SARS-CoV-2 and thus account for COVID-19 associated autoimmune endocrinopathies, such as autoimmune thyroid disease and autoimmunity against adrenals (25). In addition, vaccine-induced immunological reactions are also involved in long COVID-19, such as immune thrombotic thrombocytopenia (26). A specific endocrine event in long COVID-19 is the increased angiotensin II (ATII) levels due to the loss of ACE2 in response to SARS-CoV-2 invasion. Increased ATII can cause neutrophil accumulation, vascular hyper-permeability, pulmonary edema, the profibrotic, proapoptotic and proinflammatory signalizations in the lungs and other organs (27). Thus, increased ATII level is also an important etiology of long COVID-19 symptoms. In addition, viruses can also reduce autophagy to influence metabolism and macromolecule recycling processes while causing excessive inflammatory and autoimmune responses, as observed in long COVID-19 patients (28). By contrast, effective control of metabolic complications could prove useful therapeutic targets for combating COVID-19 (29).

### 2.3 The endocrine and metabolism disorders in long COVID sequelae

Endocrine disorders are a dramatic feature of long COVID-19. Angiotensin converse enzyme 2 (ACE2) is the primary receptor of SARS-CoV-2. The expression of ACE2 in the hypothalamus, pituitary, pancreas, thyroid, adrenal glands, testes, and ovaries makes these endocrine organs become a target for viral injury. As a result of SARS-CoV-2 attacks, there are significant decreases in hypothalamic metabolism (30), cortisol and adrenocorticotropic hormone (31), total triiodothyronine and thyroid stimulating hormone (TSH) (18), testosterone (32), and estrogen (33) levels. The extensive damage to the whole endocrine system in turn worsens immunological and metabolic disorders, such as insulin resistance and hyperglycemia in COVID-19 patients (34). In addition, histamine and histamine H2 receptor signaling is likely essential for spike protein to induce ACE2 internalization in endothelial cells and cause endothelial dysfunction (35). These endocrine problems can extend into the long COVID-19, such as hyperthyroidism, hyperglycemia and adrenal insufficiency (32). SARS-CoV-2 infection can shift cellular metabolism from oxidative phosphorylation to glycolysis and decrease ATP generation. ATP depletion contributes not only to the multiple organ failure during the acute phase, but also to elevating the susceptibility of patients with diabetes to this virus, involving immune cells and alleviating therapeutic effectiveness against SARS-CoV-2 infection (36).

According to the above changes, the sequelae of the long COVID-19 may consider as a syndrome based on damage after virus infection and neuroendocrine, immune and other disorders (37).

## 3 Therapies for the rehabilitation of long COVID-19 and the underlying mechanisms

Currently, most long COVID-19 patients are mainly treated by general practitioners. The physiological and psychological rehabilitation and treatments are considered primarily to treatment of most frequently seen symptoms of fatigue, reduced performance, poor and loss of smell and taste, or lack of concentration and so on in long COVID-19 (38).

### 3.1 General principles for the rehabilitation of long COVID-19 sequelae

Long COVID-19 requires multidisciplinary rehabilitation, including pulmonary, cardiac, sport and exercise medicine, psychological, musculoskeletal, neurological rehabilitation and general medical management (39). In the actual practice of managing long COVID-19 sequelae, psychological and physical therapies are increasingly recognized, with a focus on combating mental, respiratory and neuromuscular dysfunctions.

Six major therapeutic goals for long COVID-19 therapy based on the pathogenic mechanisms of SARS-CoV-2 should be considered (40). The first goal is determining the indicators of testing and therapy for COVID-19 patients, such as the COVID-19 molecular biomarkers and symptoms, and oxygen saturation (41). The second goal is correction of the COVID-19 patient’s hypoxia (42). The third goal is to reduce the viral load of SARS-CoV-2 by using an oral antiviral agent at early stage of COVID-19, such as molnupiravir and paxlovid pill (43). The fourth goal is to identify and address the hyperinflammation phase for those with fever and elevated C-reactive protein. Low-dose dexamethasone therapy can be an effective treatment (44). The fifth goal is to identify and address the hypercoagulability phase seen in many hospitalized COVID-19 patients with a marked increase in d-dimer and prothrombin time and a decrease in fibrinogen. Low molecular weight heparin is preferred in COVID-19 patients (45). The last goal is prophylaxis of persons without infection by using of supplements of vitamin D, vitamin C, resveratrol, and zinc (46).

These goals are essential for the prevention of long COVID-19 sequelae. For example, there was a dramatic improvement in disease severity, radiology, and pulmonary function following corticosteroids and concurrent exercise training (39). In patients who received steroid treatment, there is a mean relative increase in transfer factor following treatment of 31.6% and forced vital capacity of 9.6%, with significant symptomatic and radiological improvement (47). To correct disseminated intravascular coagulation-like phase, anticoagulation therapy with unfractionated heparin is preferred, particularly in COVID-19 patients with acute kidney injuries. Anticoagulation therapy can markedly increase d-dimer and prothrombin time with a decrease in fibrinogen. In addition, cell therapy with mesenchymal stem cells or resident lung epithelial stem/progenitor cells has been developed to prevent long-acute sequelae of COVID-19, with both pros and cons (48).

### 3.2 Psychophysical therapies for the rehabilitation of long COVID-19 and the underlying mechanisms

In general, outpatient rehabilitation of long COVID-19 includes exercise therapy, respiratory rehabilitation, photobiomodulation (PBM) therapy, psychological support such as meditation. Other rehabilitation includes essential oil smelling, activities of daily living and gait training, education, traditional Chinese medicine, and cognitive and vocational rehabilitation (49, 50). Some patients need medications to address problems such as dizziness or headaches, and others need a referral to a heart or lung specialist for further treatment. Still, others receive mental health treatment for anxiety, depression or insomnia, like physical therapy, speech therapy, rehabilitation psychology, vocational rehabilitation and social work.

#### 3.2.1 Exercise programs and physical activities.

Exercise programs and physical activities are well-known modulators of the clinical manifestations and prognosis of many chronic diseases. Regular exercise in resistance and cardiopulmonary training methods may improve many of these symptoms of long COVID-19 (51). For instance, aerobic training exercises can improve muscle strength, kinesiophobia and quality of life measures in long COVID-19 sarcopenia, particularly low-intensity aerobic training (52). The recovery of the musculoskeletal system using musculoskeletal physical therapy is able to resume a long COVID-19 woman’s daily physical activities (53). Increasing the aerobic capacity can decrease psychological problems commonly seen in people with long COVID-19 and increase immune functions by modulating the levels of glucocorticoid, oxytocin, insulin, and thyroid hormones (54). A short running exercise significantly increased the level of salivary oxytocin (55). Similarly, running wheel in mice for six weeks significantly increases oxytocin levels in the brain and serum (female only) (56).

The effect of exercise can be achieved through the following mechanisms. Exercise could stimulate the immune system and induce mitochondrial adaptations, cell generation and immune surveillance. It can also treat pulmonary complications effectively by relieving dyspnea and fatigue. Exercise can also improve cardiovascular health by enhancing mitochondrial biogenesis and function, restoring and improving vasculature, and the release of myokines from skeletal muscle. At last, stimulate brain plasticity and increases psychological well-being by improving the quality of life, controlling depression and anxiety (51).

#### 3.2.2 Oxygen therapy and essential oil smelling.

Oxygen therapy stresses on daily monitoring, non-invasive ventilation and continuous positive airways pressure delivery, pronation and longural changes to improve oxygenation, reconditioning with leg/arm cranking and exercises, initial and final patients’ functional assessment by short-physical performance battery and 1-minute sit-to-stand test to evaluate the long COVID-19 patient’s motor conditions and exercise-induced oxygen desaturation (57). Over the course of the long COVID-19 patient’s rehabilitation, exertional dyspnea, 6-min walking distance, 3-min sit-to-stand test, hyperventilation syndrome prevalence and quality of life significantly improved (58). Correspondingly, corticosteroids have been used extensively in the alleviation of acute and chronic syndromes of COVID-19 while physical therapy can decrease oxygen therapy and corticosteroid requirements during rehabilitation (59), suggesting recovery of adrenal functions and corticosteroid secretion.

Hypothalamic oxytocin neurons receive excitatory inputs from the olfactory bulbs (OBs) and the accessory OBs. Intranasally-applied oxytocin can activate oxytocin neurons by the mediation of lateral olfactory tracts (60). By contrast, olfaction deficits correlate with negative symptoms and low social drive (61), which can account for the aberrant mental activity in long COVID with anosmia. Thus, by acting on the OBs, odorants can extensively modulate brain activity, at least partially by changing hypothalamic oxytocin neuronal activity.

Studies in humans and animals have demonstrated that many odorants can increase oxytocin secretion. For example, salivary oxytocin concentrations increase significantly after exposure to aroma of certain essential oils, including lavender, neroli, jasmine absolute, roman chamomile, clary sage, and Indian sandalwood than after exposure to the control odor in postmenopausal women (62). Consistently, inhalation of lavender essential oil can ameliorate the depression-like behavior, and increase the dendritic complexity of immature neurons in the hippocampus and the subventricular zone under high corticosterone conditions (63). These effects are associated with increased oxytocin in serum (64). Rosemary extract can significantly increase central oxytocin and its receptor expressions, attenuate stress-induced changes in serum corticosterone and decrease depressive- and anxiety-like behavior in mice (65). Together with the fact that essential oils have anti-inflammatory, immunomodulatory, bronchodilatory, and antiviral properties (66), these essential oils are readily applicable for promoting the rehabilitation of long COVID directly and by increasing oxytocin secretion indirectly.

#### 3.2.3 Photobiomodulation (PBM) therapy

Recent studies on the therapeutic effects of PBM therapy in humans and animals indicate that it plays a pivotal role in long COVID-19 rehabilitation (67). LED illumination can improve skin diseases, arthritis, and osteoporosis, promote wound healing repair, participate in immune regulation (67), and increase neuroendocrine hormone secretion (68). The red and near-infrared radiation can reduce the lethality of COVID-19 (69) and this effect is associated with its reducing lung inflammation and accelerating the regeneration of damaged tissues (70). Importantly, red and near-infrared illumination through LED devices can be used to directly mobilize hormone secretion and improve pathological conditions.

So far, PBM therapy has been applied in clinical trials. It has shown good curative effect in the prevention, early treatment and the recovery of the symptoms of COVID-19 (71, 72). Clinical experimental studies have confirmed that PBM has shown good curative effects in pulmonary function rehabilitation, breathing regulation, and taste rehabilitation (73). Different studies use different wavelengths such as notably blue, red, and near-infrared light. The mechanism of PBM in treating COVID-19 is mainly manifested in the regulation of inflammatory factors, anti-oxidation and regulation of endocrine hormones (74, 75). Furthermore, topical methylene blue photodynamic virus inactivation (MB-PDI) administered in the oral and nasal cavity, combined with oral methylene blue (MB) and photobiomodulation, exerts systemic antiviral effects in patients with long-standing COVID-19 sequelae (76). The combined application of PBM and microneedle can also improve the hair loss symptoms of long-term COVID-19 syndrome (77).

#### 3.2.4 Psychological intervention and meditation.

A “Recovering from COVID” course of 7-week virtual rehabilitation takes a whole system, biopsychosocial approach to understanding COVID-19 and long-viral fatigue and is delivered by an interdisciplinary team. The course focuses on understanding long-viral fatigue, sleep optimization, nutrition, swallowing, activity management, energy conservation, stress management, breathing optimization, managing setbacks, and sign longing to appropriate resources and services. Rehabilitation is effective in reversing some of the problems faced by people living with long COVID-19 (78). In the management of anxiety in COVID-19, the general approach focuses on compassionate, similar to that during trauma or disaster, with efforts focused on instilling a sense of hope and resilience (19).

The AYUSH system proposed by the Indian government includes yoga and natural remedies to relieve post-COVID symptoms, improve lung function, improve quality of life and reduce stress (79). Mindfulness meditation is practiced widely to promote physical and mental health through cognitive performance. Observations have verified its improvements of anxiety, depression and pain scores with low-cost as well as its feasibility to practice during COVID-19 pandemic (80). It can be practiced in combination with Yogic breathing to reduce symptoms and COVID-19-associated anxiety in patients receiving dialysis. In the college population, individuals who participated in a 4-week online centering intervention showed improved of stress levels and trait mindfulness over time (81). It is also identified that mindfulness-based training can effectively mitigate the negative psychological consequences of the COVID-19 outbreak, help restore well-being in the most vulnerable individuals and have psychological well-being among nurses working for COVID-19 patients (82). All these source effects can be mediated by increasing central oxytocin actions (83).

## 4 Neuroendocrine mechanism of psychophysical therapies in long COVID-19 sequelae

These psychophysical therapies presented above are closely related to the inherent immunity (84) and metabolism as well as neuroendocrine activities in patients with long COVID-19 (85). Analyzing the mechanisms underlying psychophysical therapies for long COVID-19 reveals that changes in neuroendocrine activity underlie the effectiveness of these therapies. We propose a neuroendocrine mechanism for psychophysical therapies in the rehabilitation of long COVID-19. Specifically, we introduce the role of hormones, using oxytocin as an example, in the neuroendocrine mechanism of psychophysical therapies for the treatment of long COVID-19.

### 4.1 Oxytocin regulating anti-COVID endocrine activity

Endocrine disorders can cause physical and mental problems, whereas oxytocin can exert such functions directly and indirectly by modulating the activity of other endocrine activities. By contrast, oxytocin has antiviral effects and anti-inflammation without the long-term side-effect of corticosteroids. Thus, oxytocin can inhibit hypothalamic–pituitary–adrenal (HPA) axis activity while reserving immune-protective effects (86). Thus, the application of oxytocin at different stages of long COVID-19 in males and females can exert the effect of prevention and/or treatment of long COVID-19 sequelae. In females, oxytocin may prevent the occurrence of COVID-19 by increasing estrogen release and decreasing ACE2 expression; in males, oxytocin can reduce inflammation-evoked injury by increasing androgen release and suppressing humoral immunity (87). Although it is unclear how oxytocin affects HPA axis activity under pathological conditions, the inhibitory role of oxytocin may improve long COVID-19 sequelae of Grave’s disease that has an overproduction of thyroid hormones (88). In addition, oxytocin can reduce norepinephrine levels and thus possibly weaken stress reactions and hypertension in long COVID-19 sequelae (89). Oxytocin can increase insulin secretion by directly innervating the islets of Langerhans in the pancreas (90), maintaining β-cell adaptation reactivity (91) and increasing vagal activity (92), thereby promoting metabolic hemostasis, particularly helping control hyperglycemia in long COVID-19 (93). Metabolic disorders are important etiology of long COVID-19 sequelae alongside mitochondrial dysfunction (94). Oxytocin can compensate for tryptophan insufficiency-associated long COVID-19 sequelae (94). Together with the other reviews (95, 96), these findings indicate that oxytocin can improve long COVID-19 sequelae by regulating the activity of the endocrine system.

### 4.2 Oxytocin promoting tissue repairs and organ-specific protection

Pathogenesis underlying long COVID-19 sequelae at different organ systems vary dramatically. Oxytocin can exert protective effects through various approaches, such as promoting tissue repairs and regeneration. Oxytocin may help clear residual or hidden SARS-CoV-2 by inducing antiviral immune cell responses, as shown in the effect of Carbetocin and by blocking the interaction between SARS-CoV-2 spike protein and ACE2 (97). In pediatric patients, the long COVID-19 symptoms mainly exhibit difficulties with sustained auditory attention and divided attention while most of these patients have preexisting attention and/or mood concerns and some have elevated depression and anxiety symptoms (98). By exerting this function, oxytocin can also alleviate pain reactions in long COVID-19 (99). In addition, oxytocin can increase the expression of pulmonary surfactants, promote angiogenesis and regeneration of infarcted cardiomyocytes, prevent atherosclerosis and coronary artery disease, and many other complications associated with long COVID-19, thereby becoming a strong candidate hormone to improve long COVID-19 sequelae (95). Immune system dysregulation causes tissue damages through hyperinflammation, cytokine storm syndrome, and immune-mediated multi-system damage. It is well known that Oxytocin treatment can weaken the neuroinflammatory process (100). Oxytocin inhibits LPS-induced inflammation and attenuates microglial activation in LPS-treated mice (101), which provides long-term neuroprotection and likely alleviates brain neurological symptoms in long COVID-19 (102).

## 5 Conclusions and perspectives

Long COVID-19 is the sequelae in COVID-19 survivors and significantly influences the healthcare system (103). However, current understandings of the pathogenesis and rehabilitation strategy remain quite limited (104). By analyzing the underlying mechanisms of psychophysical therapies for long COVID-19, a common involvement of neuroendocrine activity is revealed (**Figure 1**). In view of the diverse clinical manifestations of COVID-19 and long COVID-19, it is recommended that individualized treatment should be considered in both inpatient treatment (community hospitals and general hospitals) and out-of-hospital treatment (family treatment) during psychophysical therapies (105). At the same time, it is recommended to use ICF (International Classification of Functioning, Disability and Health), a method proposed by WHO in 1996 to describe the health status of patients based on biological, psychological and social perspectives, to accurately evaluate the effect of psychophysical therapy and choose physical psychotherapy methods on the sequelae of long COVID-19.

## Author contributions

QM and WS contributed equally to this work and wrote the first manuscript. QM, WS, LC, FMZ, and YFW contributed to the critical revision of the manuscript. FMZ, and YFW conceived the study and edited the text. All authors approved the submitted version.
